# *Dolosigranulum pigrum* Modulates Immunity against SARS-CoV-2 in Respiratory Epithelial Cells

**DOI:** 10.3390/pathogens10060634

**Published:** 2021-05-21

**Authors:** Md. Aminul Islam, Leonardo Albarracin, Vyacheslav Melnikov, Bruno G. N. Andrade, Rafael R. C. Cuadrat, Haruki Kitazawa, Julio Villena

**Affiliations:** 1 Food and Feed Immunology Group, Laboratory of Animal Food Function, Graduate School of Agricultural Science, Tohoku University, Sendai 981-8555, Japan; aminul.vmed@bau.edu.bd (M.A.I.); lalbarracin@herrera.unt.edu.ar (L.A.); 2 Department of Medicine, Faculty of Veterinary Science, Bangladesh Agricultural University, Mymensingh 2202, Bangladesh; 3 Laboratory of Immunobiotechnology, Reference Centre for Lactobacilli (CERELA-CONICET), Tucumán 4000, Argentina; 4 Gabrichevsky Research Institute for Epidemiology and Microbiology, 125212 Moscow, Russia; goutch@mail.ru; 5 AdaptCentre, Munster Technological University (MTU), T12 P928 Cork, Ireland; Bruno.Andrade@cit.ie; 6 Max-Delbrück-Center for Molecular Medicine in the Helmholtz Association (MDC), Berlin Institute for Medical Systems Biology (BIMSB), 13125 Berlin, Germany; rafaelcuadrat@gmail.com; 7 Department of Molecular Epidemiology, German Institute of Human Nutrition Potsdam-Rehbrücke, 14558 Nuthetal, Germany; 8 Livestock Immunology Unit, International Education and Research Center for Food and Agricultural Immunology (CFAI), Graduate School of Agricultural Science, Tohoku University, Sendai 981-8555, Japan

**Keywords:** respiratory commensal bacteria, respiratory epithelial cells, next generation probiotics, *Dolosigranulum pigrum*, SARS-CoV-2, COVID-19, coronavirus

## Abstract

In a previous work, we demonstrated that nasally administered *Dolosigranulum pigrum* 040417 beneficially modulated the respiratory innate immune response triggered by the activation of Toll-like receptor 3 (TLR3) and improved protection against Respiratory Syncytial Virus (RSV) in mice. In this work, we aimed to evaluate the immunomodulatory effects of *D. pigrum* 040417 in human respiratory epithelial cells and the potential ability of this immunobiotic bacterium to increase the protection against Acute Respiratory Syndrome Coronavirus 2 (SARS-CoV-2). The respiratory commensal bacterium *D. pigrum* 040417 differentially modulated the production of IFN-β, IL-6, CXCL8, CCL5 and CXCL10 in the culture supernatants of Calu-3 cells stimulated with poly(I:C) or challenged with SARS-CoV-2. The differential cytokine profile induced by the 040417 strain was associated with a significant reduction in viral replication and cellular damage after coronavirus infection. Of note, *D. pigrum* 030918 was not able to modify the resistance of Calu-3 cells to SARS-CoV-2 infection, indicating a strain-specific immunomodulatory effect for respiratory commensal bacteria. The findings of this work improve our understanding of the immunological mechanisms involved in the modulation of respiratory immunity induced by respiratory commensal bacteria, by demonstrating their specific effect on respiratory epithelial cells. In addition, the results suggest that particular strains such as *D. pigrum* 040417 could be used as a promising alternative for combating SARS-CoV-2 and reducing the severity of COVID-19.

## 1. Introduction

Severe Acute Respiratory Syndrome Virus 2 (SARS-CoV-2) is the etiological agent of the coronavirus disease 19 (COVID-19) pandemic. SARS-CoV-2 receptor recognition and attachment are initiated via interactions between the viral S protein and the human angiotensin-converting enzyme 2 (ACE2) expressed by susceptible cells such as respiratory epithelial cells of the airways and the lung parenchyma [1]. Variable clinical manifestations have been reported for COVID-19, ranging from mild respiratory symptoms to acute lung injury and severe acute respiratory distress syndrome [2]. It was shown that in addition to the cytopathic effect induced by the virus, the respiratory alterations caused by SARS-CoV-2 are mediated by an aggressive inflammatory response that significantly contributes to damage in the respiratory tissues. The impaired responses of type I and type III interferons (IFNs) and antiviral factors have been associated with more severe COVID-19 cases [3,4,5]. In addition, it was reported that the replication of SARS-CoV-2 induces extensive death in epithelial cells, and causes the release of pro-inflammatory cytokines/chemokines and the recruitment of inflammatory cells into the respiratory tract [6]. Of note, it was observed that the reduced production of IFN-β in patients with COVID-19 is accompanied by elevated secretion of interleukin (IL)-6, chemokine (C-C motif) ligand 2 (CCL2), CCL5, CCL8, chemokine (C-X-C motif) ligand 8 (CXCL8), CXCL9, CXCL16, and CXCL2, which contribute to aggravating COVID-19 pathology [7,8] through the recruitment of inflammatory monocytes and neutrophils into the respiratory tract [6].

The clinical characteristics and the cellular and molecular events involved in the pathophysiology of COVID-19 have been extensively investigated, and as was mentioned above, great importance has been given to the interaction between SARS-CoV-2 and the host’s immune system in the outcome of the disease. However, recent studies indicate that a third actor could be important in the progression and severity of COVID-19: the respiratory microbiota. In this regard, some works have studied the potential impact of the respiratory microbiota on the severity and outcome of SARS-CoV-2 infection [9,10,11]. A study investigated the nasopharynx microbial community of patients that developed different severity levels of COVID-19, including mild, moderate and critical cases [10]. The work found that *Prevotella* spp. and *Veillonella* spp. were significantly more abundant in patients that presented more severe COVID-19. Interestingly, the work also suggested that *Dolosigranulum* spp. might protect against the development of severe COVID-19. These findings have been recently supported by a report describing lower abundances of *Dolosigranulum pigrum* among young adults that developed symptoms in the context of SARS-CoV-2 infection [11]. Although this preliminary evidence does not allow us to conclusively state that differences in microbiota composition are the cause of the different COVID-19 severities, the data suggest that the role of the respiratory microbiota in SARS-CoV-2 infection warrants further investigation.

The potential beneficial effect of *D. pigrum* against respiratory infections was recently reported by our group using mice models of pneumococcal and Respiratory Syncytial Virus (RSV) infections [12]. In our hands, the nasal administration of *D. pigrum* 040417 to mice prior to the challenge with the Toll-like receptor 3 (TLR3) agonist polyinosinic:polycytidylic acid poly(I:C) or RSV differentially modulated the respiratory innate immune response, allowing improved viral clearance and reduced inflammatory-mediated lung damage. Of note, the ability of *D. pigrum* to beneficially modulate the respiratory innate immune response was a strain-dependent characteristic, since other strains such as *D. pigrum* 030918 were not able to achieve this benefit [12].

All in all, these data indicate that immunomodulatory members of the respiratory microbiota could be capable of beneficially regulating the immune response against SARS-CoV-2. In this work, we aimed to evaluate whether the immunomodulatory strain *D. pigrum* 040417 was able to differentially modulate cytokine production in the culture supernatants of Calu-3 cells stimulated with poly(I:C) or challenged with SARS-CoV-2, and in this way, to reduce viral replication.

## 2. Materials and Methods

### 2.1. Respiratory Commensal Bacteria

*Dolosigranulum pigrum* 040417 and *D. pigrum* 030918 were cultured at 37 °C for 18 h (late log phase) in trypticase soy broth. Bacteria suspensions were prepared as previously described [12]. Briefly, the bacteria were harvested by centrifugation at 3000× *g* for 10 min, washed three times with sterile 0.01 M phosphate buffer saline (PBS, pH 7.2), and suspended in sterile PBS.

### 2.2. Cell Cultures

Calu-3 cells, a human lung epithelial cell line originating from a human pulmonary adenocarcinoma (HTB-55, American Type Culture Collection (ATCC), Manassas, VA, USA), was grown in Dulbecco’s modified Eagle medium (DMEM) supplemented with 20% fetal bovine serum (FBS) and 1% non-essential amino acid solution (Gibco, Grand Island, NY, United States). For antiviral assays, the FBS was reduced to 10% and 1% penicillin/streptomycin (Gibco) was added to the medium. Vero 76 cells, from an African green monkey kidney epithelial cell line (ATCC CRL-1587), were grown in minimal essential medium (MEM) supplemented with 10% of FBS. For antiviral assays, the FBS was reduced to 2%. The cells were incubated at 37 °C in a humidified incubator in an atmosphere with 5% CO_2_.

### 2.3. Cytokine Concentrations in Culture Supernatants

Calu-3 cells were cultured as described above and seeded in 6-well plates at a density of 2 × 10^6^ cells/well. For the evaluation of the effect of respiratory commensal bacteria, 1 mL of DMEM containing the different *D. pigrum* strains (5 × 10^7^ cells/mL) was added to Calu-3 cells monolayers. The cells were further incubated for 24 h at 37 °C, 5% CO_2_. Bacteria were removed by washing with PBS and the respiratory epithelial cells were stimulated with 15 μg/mL of the synthetic double-stranded RNA (dsRNA) poly(I:C) (Sigma-Aldrich). Before the challenge with poly(I:C) (basal levels) and 48 h after the activation of TLR3, culture supernatants were collected for the determination of cytokines by available enzyme-linked immunosorbent assay (ELISA) technique kits.

The concentrations of tumor necrosis factor (TNF)-α (sensitivity: 6.23 pg/mL), interferon (IFN)-β (sensitivity: 0.7 pg/mL), interleukin (IL)-1β (sensitivity: 1 pg/mL), IL-6 (sensitivity: 0.7 pg/mL), chemokine (C-C motif) ligand 5 (CCL5 or RANTES) (sensitivity: 6.6 pg/mL), chemokine (C-X-C motif) ligand 8 (CXCL8 or IL-8) (sensitivity: 7.5 pg/mL) and CXCL10 (or interferon gamma-induced protein 10, IP-10) (sensitivity: 4.46 pg/mL) were measured with commercial ELISA kits following the manufacturer’s recommendations (R&D Systems, MN, USA).

### 2.4. SARS-CoV-2 Infection

A SARS-CoV-2 clinical isolate (hCoV-19/USA/VA/2020) was passaged twice in Vero 76 cells to create working stocks. For experiments with SARS-CoV-2 infection, Calu-3 2B4, a subpopulation of Calu-3 cells with high ACE2 expression, was used. The Calu-3 2B4 cells were obtained by sorting with an ACE2 antibody as described previously [13]. The Calu-3 2B4 cells were stimulated with 1 mL of DMEM containing the different *D. pigrum* strains (5 × 10^7^ cells/mL) for 24 h at 37 °C, 5% CO_2_. The respiratory commensal bacteria were removed by PBS washing and then Calu-3 2B4 cells were challenged with SARS-CoV-2 (MOI 0.1 PFU) at 37 °C. After 30 min, the cells were washed once with PBS to remove any unbound virus, and the cells were incubated for 48 or 72 h at 37 °C, 5% CO_2_.

Infectious SARS-CoV-2 plaque-forming units (PFU) were quantified by plaque titration on Vero 76 cells, as described elsewhere [14,15,16], with minor modifications. Vero 76 monolayers were seeded in 24-well plates, washed with PBS, incubated with serial dilutions of SARS-CoV-2-containing cell culture supernatants in duplicates, and overlaid with 1.2% Avicel in DMEM. After 72 h, the cells were fixed with 6% formalin and visualized by crystal violet staining.

The cytosolic enzyme lactate dehydrogenase (LDH) is released upon damage to the plasma membrane and can be measured in the supernatant of Calu-3 2B4 cells as an indicator of cellular toxicity. The LDH levels in the supernatants of Calu-3 2B4 cells were measured by the LDH assay kit (Weiner Lab). The concentrations of TNF-α, IFN-β, IL-1β, IL-6, CCL5, CXCL8 and CXCL10 were measured by ELISA kits as described above.

All the experiments were conducted in Biosafety Level-3 (BSL-3) facilities according to the biosafety guidance related to COVID-19 [17,18].

### 2.5. Statistical Analysis

Experiments were performed in triplicate and the results are expressed as mean ± standard deviation (SD). Normally distributed data were tested by 2-way ANOVA. Tukey’s test (for pairwise comparisons of the means) or the Fisher’s least significant difference (LSD) test (for multi-comparison) were used to evaluate the differences among groups. Differences were considered significant at *p* < 0.05 or *p* < 0.01.

## 3. Results

**Figure 1:**
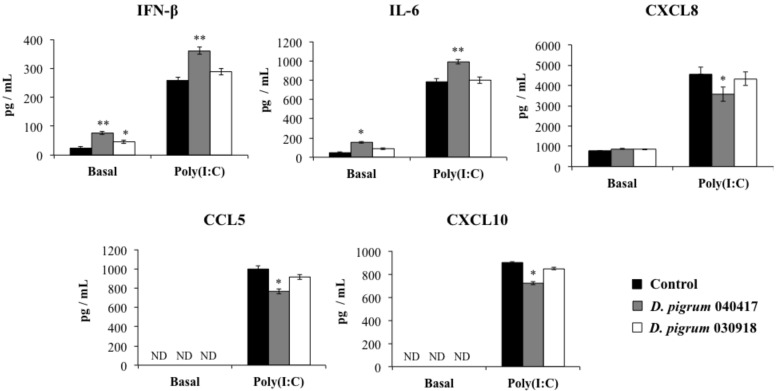
Effect of *Dolosigranulum pigrum* strains on the innate immune response of human respiratory epithelial cells triggered by the activation of Toll-like receptor 3 (TLR3). Calu-3 cells were stimulated with *D. pigrum* 040417 or 030918 (5 × 10^7^ cells/mL) for 24 h and then challenged with poly(I:C) for 48 h. Calu-3 cells with no bacterial treatment and stimulated with poly(I:C) were used as controls. Before (basal groups) and after (poly(I:C) groups) the activation of TLR3, culture supernatants were collected for the determination of cytokines by ELISA. ND: not detected. The results represent data from three independent experiments. Significant difference when compared to control at the same time point: * *p* < 0.05 or ** *p* < 0.01.

We first evaluated whether the stimulation of respiratory epithelial cells with *D. pigrum* modulated their cytokine profile. The treatment with *Dolosigranulum* strains did not induce visible adverse effects in epithelial cells or modify the levels of LDH in culture supernatants (Supplementary Figure S1). As shown in Figure 1 (basal), the stimulation of cells with *D. pigrum* 040417 significantly increased the production of IFN-β and IL-6, while no modifications were observed in the production of CXCL8. This effect was strain-dependent, since the stimulation of Calu-3 cells with *D. pigrum* 030918 did not induce modifications in the levels of IL-6 and CXCL8. In addition, although the 030918 strain increased the production of IFN-β when compared to controls, the values did not reach the levels found in cells stimulated with the 040417 strain (Figure 1). Basal levels of CCL5, CXCL10 (Figure 1), TNF and IL-1β (data not shown) were not detected in culture supernatants by using the ELISA kits.

We next evaluated the influence of respiratory commensal bacteria on the response of Calu-3 cells to a challenge with the TLR3 agonist poly(I:C). The stimulation of respiratory epithelial cells with the synthetic dsRNA increased the levels of IFN-β, IL-6, and CXCL8 by 4- to 5-fold in controls and cells pre-treated with both strains. Furthermore, detectable levels of CCL5 and CXCL10 were found in culture supernatants of cells challenged with poly(I:C) (Figure 1). The pre-treatment of Calu-3 cells with *D. pigrum* 040417 significantly increased the production of IFN-β and IL-6 and reduced the levels of CXCL8, CCL5 and CXCL10 when compared to control cells. In contrast, *D. pigrum* 030918 was not able to significantly change the levels of the evaluated cytokines (Figure 1). TNF and IL-1β were not detected in culture supernatants of cells stimulated with poly(I:C) by using the ELISA kits (data not shown). This is in agreement with Chen et al. [19], who found no detectable levels of both cytokines after the challenge of Calu-3 cells with TLR3 or TLR7 agonists.

**Figure 2:**
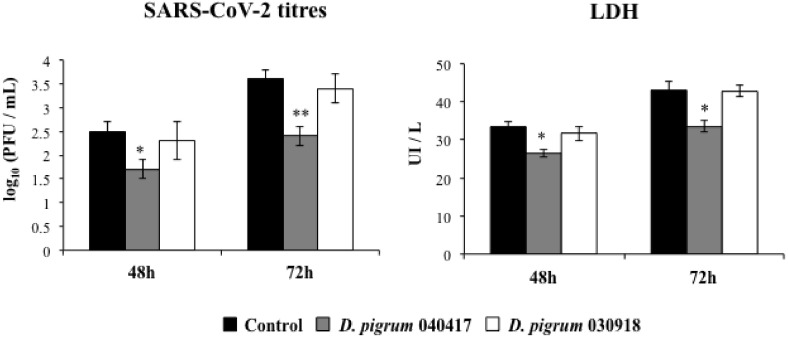
Effect of *Dolosigranulum pigrum* strains on the resistance of human respiratory epithelial cells to infection with severe acute respiratory syndrome virus 2 (SARS-CoV-2). Calu-3 2B4 cells were stimulated with *D. pigrum* 040417 or 030918 (5 × 10^7^ cells/mL) for 24 h and then challenged with SARS-CoV-2. Calu-3 2B4 cells with no bacterial treatment and challenged with the virus were used as controls. SARS-CoV-2 titers and LDH levels in culture supernatants were determined at hours 48 and 72 post-infection. The results represent data from three independent experiments. Significant difference when compared to the control at the same time point: * *p* < 0.05 or ** *p* < 0.01.

Coronaviruses are enveloped viruses with positive-sense single-stranded RNA (ssRNA) genomes. These viruses produce double-stranded RNA (dsRNA) molecules during genome replication and mRNA transcription [20]. In this regard, it was reported recently that SARS-CoV-2 is sensed by the antiviral systems that detect dsRNA in respiratory epithelial cells [21]. Then, considering the ability of *D. pigrum* 040417 to differentially modulate the cytokine profile induced by poly(I:C) stimulation in respiratory epithelial cells, we hypothesized that this bacterium may influence the replication of SARS-CoV-2 in Calu-3 cells. To demonstrate this hypothesis, the highly ACE2-expressing Calu-3 2B4 cells were stimulated with *D. pigrum* 040417 or 030918 and subsequently challenged with SARS-CoV-2. Viral titers and LDH as a marker of cellular damage were evaluated at 48 and 72 h post-infection (Figure 2). We evaluated the SARS-CoV-2 replication in Calu-3 2B4 cells at 48 and 72 h post-infection, taking into consideration that it was reported that the replication kinetics of this virus in these cells is slower than that observed in Vero 76 cells [21]. The virus was detected at both time points, with higher titers at hour 72. Consistent with these results, higher levels of LDH were detected in the culture supernatants of infected cells at hour 72 than at hour 48. Of note, the pre-treatment of respiratory epithelial cells with *D. pigrum* 040417 significantly reduced SARS-CoV-2 replication and LDH values at hours 48 and 72 when compared to control cells. In contrast, *D. pigrum* 030918 was not able to improve the resistance of Calu-3 2B4 cells against coronavirus infection (Figure 2).

**Figure 3:**
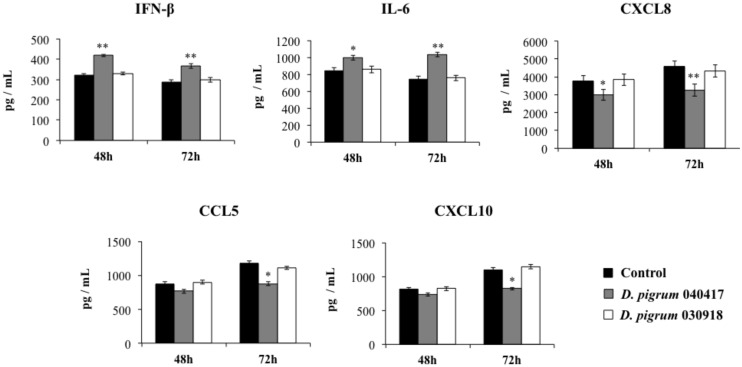
Effect of *Dolosigranulum pigrum* strains on the innate immune response of human respiratory epithelial cells triggered by infection with severe acute respiratory syndrome virus 2 (SARS-CoV-2). Calu-3 2B4 cells were stimulated with *D. pigrum* 040417 or 030918 (5 × 10^7^ cells/mL) for 24 h and then challenged with SARS-CoV-2. Calu-3 2B4 cells with no bacterial treatment and challenged with the virus were used as controls. Cytokines levels in culture supernatants were determined by ELISA at hours 48 and 72 post-infection. The results represent data from three independent experiments. Significant difference when compared to the control at the same time point: * *p* < 0.05 or ** *p* < 0.01.

In order to evaluate whether the enhanced resistance of respiratory epithelial cells to SARS-CoV-2 challenge was associated with a different cytokine profile, we further evaluated the concentrations of IFN-β, IL-6, CXCL8, CCL5 and CXCL10 in the culture supernatants of infected cells (Figure 3). The coronavirus infection triggered the production of all the cytokines evaluated. While the levels of IFN-β and IL-6 reached values that were similar at the two time points evaluated, the production of CXCL8, CCL5 and CXCL10 was higher at hour 48 than hour 72. Interestingly, the pre-treatment of respiratory epithelial cells with *D. pigrum* 040417 significantly increased the levels of both IFN-β and IL-6 induced by the SARS-CoV-2 infection. In addition, the 040417 strain reduced the production of CXCL8 at the two times points evaluated and diminished the levels of CCL5 and CXCL10 at hour 72 post-infection (Figure 3). *D. pigrum* 030918 was not able to modify the cytokine response of Calu-3 2B4 cells to the SARS-CoV-2 infection (Figure 3).

## 4. Discussion

Respiratory commensal bacteria are able to interact with different types of cells in the respiratory tract [22,23], including respiratory epithelial cells [23] as well as dendritic cells (DCs) and alveolar macrophages [22]. Accumulating evidence has emerged demonstrating that these respiratory microbiota–host interactions play key roles in the pathogenesis of respiratory infections. In fact, several lines of evidence suggested that some species of bacteria, including *Corynebacterium* spp. and *Dolosigranulum* spp., play a protective role in the respiratory tract [24], considering that the levels of both types of bacteria were correlated with an improved resistance against bacterial [25,26] and viral infections [27,28,29]. In line with these reports, we demonstrated that the nasal administration of *D. pigrum* 040417 to mice beneficially regulated the respiratory innate immune response triggered by the activation of TLR2 or TLR3, and enhanced the resistance to *Streptococcus pneumoniae* and RSV infections [12]. The treatment of animals with the 040417 strain significantly reduced lung damage, which was mediated by the inflammatory response triggered by the respiratory pathogens. In addition, we were able to demonstrate that the improved protection against the pathogens induced by *D. pigrum* 040417 was related to the ability of this bacterium to stimulate CD11c^+^CD11b^high^MHCII^+^ and CD11c^+^CD103^+^MHCII^+^ DCs and improve the response mediated by respiratory CD3^+^CD4^+^IFN-γ^+^ T cells [12]. In this work, we extend and complement those previous findings by demonstrating for the first time that *D. pigrum* 040417 is also capable of modulating the response of respiratory epithelial cells to stimulation with poly(I:C). It is tempting to speculate that respiratory commensal bacteria like *D. pigrum* 040417 establish complex molecular interactions with respiratory epithelial cells, modifying their immunobiology and increasing their defense functions, as was described for beneficial microorganisms that interact with the epithelial cells of the intestinal mucosa [30,31,32]. Of note, Calu-3 cells pre-treated with *D. pigrum* 040417 produced higher levels of IFN-β and IL-6 and lower levels of CXCL8, CCL5 and CXCL10 in response to poly(I:C) stimulation when compared to control cells. These results are in line with our previous in vivo experiments demonstrating that mice nasally treated with the 040417 strain had improved levels of IFN-β, reduced concentrations of inflammatory cytokines and chemokines, and diminished numbers of neutrophils in the respiratory tract after poly(I:C) or RSV challenges [12]. It should be noted that one limitation of our study is that cell lines may not entirely reflect the in vivo response of epithelial cells to bacterial or viral stimulations. Therefore, further studies using human primary respiratory epithelial cells would be of value to further demonstrate the protective effect of *D. pigrum* 040417.

We also demonstrated here for the first time that an immunomodulatory respiratory commensal bacterium is able to differentially modulate the innate immune response of respiratory epithelial cells triggered by SARS-CoV-2 infection. Moreover, we showed that SARS-CoV-2 replication in respiratory epithelial cells pretreated with *D. pigrum* 040417 was significantly diminished. Our results are in line with recently published works that studied the potential effects of the respiratory microbiota on the severity and outcome of SARS-CoV-2 infection [9,10,11]. The comparison of the bacterial communities of the nasopharynx in uninfected and SARS-CoV-2-infected patients did not find any differences between the groups [9]. However, it should be noted that in that work, only patients with mild COVID-19 that did not develop pneumonia were included to evaluate the differences in bacterial richness and diversity, in comparison to uninfected patients. In contrast, Ventero et al. [10] studied the microbiota of the nasopharyngeal tract in COVID-19 patients with different degrees of severity by using advanced techniques of molecular microbiology, and revealed a differential abundance in several bacterial taxa among the COVID-19 cases. The metagenomic study found that more than sixty operational taxonomic units (OTUs) were exclusive to SARS-CoV-2-positive patients, most of these microorganisms being members of the phylum *Bacteroidota* and *Firmicutes*. Furthermore, *Prevotella* spp. was the most common genera among the OTUs found exclusively in COVID-19-positive patients with severe outcomes. Of note, *Dolosigranulum* spp. was identified in the group of patients with mild COVID-19 that did not need hospitalization, and displayed a negative association with *Prevotella* spp. A recent study identified nasopharyngeal microbiome alterations, including lower abundances of *D. pigrum* among children, adolescents, and young adults, which were associated with the presence of respiratory symptoms in the context of SARS-CoV-2 infection [11].

Our results suggest that *D. pigrum* 040417 would improve the resistance of respiratory epithelial cells to SARS-CoV-2 by modulating the innate immune response triggered by the cellular system recognizing viral dsRNA molecules. The detection of dsRNA from coronaviruses by the host’s pattern recognition receptors leads to the production of type I and type III IFNs, the expression of IFN-stimulated genes (ISGs) [33], and the activation of the protein kinase R (PKR) [34] and oligoadenylate synthetase–ribonuclease L (OAS-RNaseL) antiviral systems [35]. It was reported that coronaviruses are efficient in evading these host antiviral IFN signaling, PKR and OAS-RNaseL systems, and that the dysregulated antiviral responses are associated with severe infections [3,4,5]. The dsRNA-induced pathway activation during SARS-CoV-2 infection in Calu-3 cells was recently investigated by Li et al. [21]. It was shown that SARS-CoV-2 can trigger the expression of IFNs and ISGs (particularly OAS2). It was also found that SARS-CoV-2 did not activate dsRNA-induced pathway responses as robustly as alphavirus Sindbis virus (SINV) in respiratory epithelial cells [21,35]. However, the expression of these factors and the phosphorylation of STAT1 were significantly higher when compared to those induced by MERS-CoV challenge. These data suggest that SARS-CoV-2 may not antagonize dsRNA pathways as efficiently as MERS-CoV, and therefore the modulation of the IFN-signaling pathway during the earlier stage of infections is an interesting alternative to reduce viral replication. Then, the early improved production of IFN-β induced by *D. pigrum* 040417 in Calu-3 cells (and probably also ISGs) would be associated with the lower viral replication.

It was reported that during initial stages of SARS-CoV-2 infection, there is a weak and delayed IFN response in some individuals. Moreover, the IFN produced later during the course of the infection was associated with the induction of a strong inflammatory response and resulting immunopathology [36]. Therefore, the ability of the host to both control SARS-CoV-2 replication and to regulate the inflammatory response determines the outcome of COVID-19. Transcriptomic studies performed in Calu-3 cells infected with SARS-CoV-2 described an upregulation of genes mostly enriched in inflammatory-related pathways, including the nuclear factor kappa B (NF-kB) signaling and cytokine-mediated signaling pathways, suggesting that the viral challenge induces inflammatory responses in an early stage of infection [37]. Interestingly, among the upregulated cytokines/chemokines induced by SARS-CoV-2 challenge, the authors found enhanced expressions of CXCL8, CSF3, CSF2, CXCL10, and CCL2 in infected Calu-3 cells. These cytokines/chemokines were reported to exhibit substantially elevated serum levels in patients with severe clinical symptoms of COVID-19 [7,8], and deregulated respiratory and systemic inflammation [6]. In line with these findings, we observed here that the challenge of Calu-3 cells with SARS-CoV-2 significantly increased the production of CXCL8, CCL5, and CXCL10, which are chemoattractants for neutrophils and T cells. Since epithelial cells play an important role in the recruitment and activation of inflammatory cells in the mucosal tissues [23], it is tempting to speculate that the reduction of CXCL8, CCL5, and CXCL10 production induced by *D. pigrum* 040417 treatment in SARS-CoV-2-inefected Calu-3 cells would contribute in vivo to controlling inflammatory damage. Interestingly, we have reported that the nasal treatment of mice with the 040417 strain increased lung CD3^+^CD4^+^IL-10^+^ T cells after RSV infection, contributing to the control of inflammation-mediated lung tissue damage [12]. Thus, the potential anti-inflammatory effects of *D. pigrum* 040417 in the context of SARS-CoV-2 infection warrants further investigation.

The beneficial effects of intestinal microbes on mucosal immunity have been shown to be a strain-specific property, since the immunomodulatory activity of one strain cannot be extrapolated to others even of the same species [31,38]. Our previous work demonstrated for the first time the strain-dependent ability of respiratory commensal bacteria of the species *C. pseudodiphtheriticum* and *D. pigrum* to beneficially modulate respiratory immunity [12]. The results obtained here in cell cultures are in line with our previous in vivo findings. While *D. pigrum* 040417 differentially modulated the response of respiratory epithelial cells to poly(I:C) challenge or SARS-CoV-2 infection, the strain *D. pigrum* 030918 was not able to induce modifications in the immune responses of Calu-3 cells. The fact that the beneficial effects of respiratory commensal bacteria are a strain-dependent characteristic is of high importance when selecting the most efficient candidates. In this regard, further studies with a higher number of *D. pigrum* strains (or other beneficial respiratory commensal bacteria such as *C. pseudodiphtheriticum*) could help us in finding bacteria that could be more efficient than the 040417 strain in increasing resistance to SARS-CoV-2 infection. An interesting source of these strains could be patients infected with the coronavirus who do not develop the disease or have mild symptoms. This is an interesting study that we intend to perform in the immediate future.

Of note, *D. pigrum* 040417 was capable to increase the resistance of Calu-3 cells to SARS-CoV-2, but this immunobiotic commensal bacterium was not able to completely avoid the infection with the respiratory pathogen. It was suggested that the members of the respiratory microbiota are able to modulate each other’s functions. The frequent co-occurrence of *Dolosigranulum* spp. and *Corynebacterium* spp. in the respiratory mucosa has been attributed to the ability of *Dolosigranulum* spp. to facilitate the expansion of *Corynebacterium* spp. [25]. Interestingly, Ventero et al. [10] found that *Corynebacterium* was positively associated with *Dolosigranulum* in the co-abundance network analysis, indicating that in mild symptomatic SARS-CoV-2-infected patients, those two taxa form a consortium that might protect against COVID-19 development. This co-abundance connection was lost in severe cases of COVID-19. In contrast, Hurst et al. [11] suggested that *Corynebacterium* spp. were more abundant in SARS-CoV-2-infected patients with respiratory symptoms compared to infected individuals without respiratory symptoms. It should be mentioned that the *Corynebacterium* species suggested to be negatively associated with the outcome of COVID-19 were *C. accolens*, *C. macginleyi* and *C. tuberculostearicum*. To evaluate whether the co-administration of *D. pigrum* 040417 and strains of the species *C. pseudodiphtheriticum, C. accolens*, *C. macginleyi* or *C. tuberculostearicum* improves or impairs the resistance of respiratory epithelial cells to SARS-CoV-2 infection would also be an interesting topic for research.

## 5. Conclusions

To date, studies of the respiratory microbiota and SARS-CoV-2 infection have been conducted among small cohorts of patients with different degrees of COVID-19 severity [9,10,11]. In addition, the samples for the evaluation of the respiratory microbiota were collected at a single time point after SARS-CoV-2 infection, and studies did not evaluate samples prior to the viral challenge. Therefore, the research works were unable to determine if the differences observed in the respiratory microbiota of COVID-19 patients preceded, or were the consequence of, the SARS-CoV-2 infection. The results presented in this work provide evidence that supports the first hypothesis postulating that the presence of beneficial bacteria in the respiratory tract may favorably influence the response to SARS-CoV-2 infection. Although further mechanistic and in vivo studies are necessary to propose specific strains of beneficial respiratory commensal bacteria, such as *D. pigrum* 040417, for the prevention or treatment of the respiratory infection caused by SARS-CoV-2, this work represents an important first step in that direction.

## Data Availability

All the data related to this project are presented here.

## Supplementary Materials

The following are available online at https://www.mdpi.com/article/10.3390/pathogens10060634/s1, Supplementary Figure S1. Effect of *Dolosigranulum pigrum* strains on human respiratory epithelial cells. Calu-3 cells were stimulated with *D. pigrum* 040417 or 030918 (5 × 10^7^ cells/mL) for 24 h. Calu-3 cells with no bacterial treatment were used as controls. LDH levels in culture supernatants were determined to evaluate cytotoxicity. The results represent data from three independent experiments.
