# Supplementary figures and images for: Dolosigranulum pigrum Modulates Immunity against SARS-CoV-2 in Respiratory Epithelial Cells
Source: Pathogens. 2021 May 21;10(6):634. doi: 10.3390/pathogens10060634 (PMC8224358; doi:10.3390/pathogens10060634)

## Slide 1
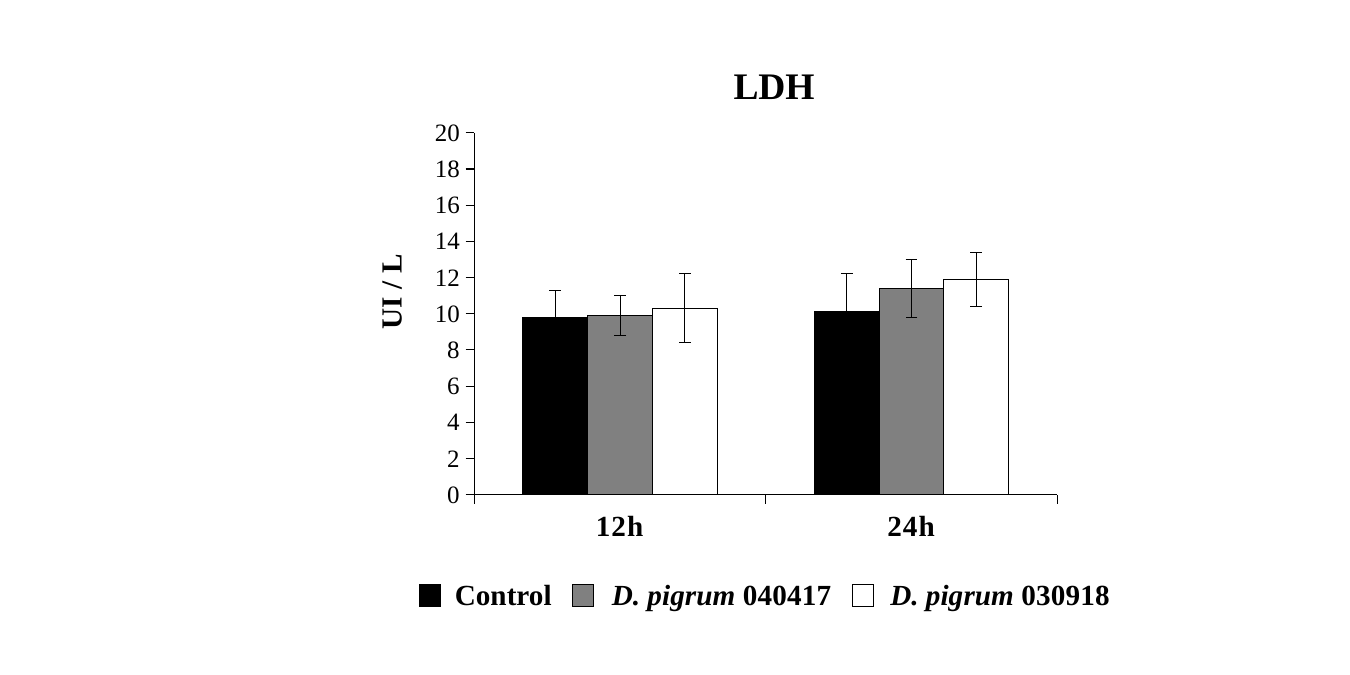

LDH
### Chart
| Category | Control | DP040417 | DP30918 |
|---|---|---|---|
| 12h | 9.8 | 9.9 | 10.3 |
| 24h | 10.1 | 11.4 | 11.9 |UI / L
Control
 D. pigrum 030918
 D. pigrum 040417

Supplement: Supplementary file 1 [file pathogens-10-00634-s001.zip › pathogens-1203262-supplementary.pptx]
